# Application of a combined predictive model based on lung ultrasound score trajectory changes in deciding mechanical ventilator weaning for neonatal respiratory distress syndrome: a retrospective study

**DOI:** 10.3389/fmed.2026.1764757

**Published:** 2026-03-11

**Authors:** Li Jiang, Fan Li, Lili Hong, Xiaoling Yang, Lu Xiao, Lili Ke, Ling Ma, Fengxi Chen, Zhigui Zhang, Linhao Ran

**Affiliations:** 1 Department of Ultrasound, Banan Hospital of Chongqing Medical University, Chongqing, China; 2 Department of Ultrasound, Children’s Hospital of Chongqing Medical University, Chongqing, China; 3 Department of Neonatal, Banan Hospital of Chongqing Medical University, Chongqing, China; 4 Department of Radiology, Banan Hospital of Chongqing Medical University, Chongqing, China; 5 Department of Nuclear Medicine & Minimally Invasive Interventional, Banan Hospital of Chongqing Medical University, Chongqing, China

**Keywords:** lung ultrasound score trajectory, mechanical ventilation, neonatal respiratory distress syndrome, nomogram, predictive model

## Abstract

**Objective:**

Neonatal respiratory distress syndrome (NRDS) often requires mechanical ventilation, and accurate prediction of extubation timing is crucial.

**Methods:**

A retrospective cohort of neonates with NRDS who underwent mechanical ventilation between January 2020 and December 2024 was included. Patients were divided into success and failure groups according to reintubation within 48 h post-extubation. A predictive model was constructed by integrating LUS trajectory changes, gestational age (GA), partial pressure of oxygen (PaO_2_), and oxygenation index (OI), with multivariate analysis performed to evaluate predictive ability.

**Results:**

The results demonstrated that LUS trajectory (LUS-high: OR = 24.099, LUS-medium: OR = 6.676,), GA (OR = 0.759), PaO_2_ (OR = 0.964), and OI (OR = 1.409) were significant predictors of extubation outcomes. The nomogram incorporating these four factors exhibited an area under the curve (AUC) of 0.914. The Hosmer–Lemeshow test indicated good model fit (*p* = 0.624), and the calibration curve closely approximated the ideal diagonal. Additionally, decision curve analysis revealed superior net benefit for the model. The internal validation cohort confirmed the reliability of the predictive nomogram.

**Conclusion:**

Dynamic LUS assessment, combined with GA, PaO_2_, and OI, effectively predicts extubation outcomes in preterm neonates with NRDS undergoing mechanical ventilation. The model could aid in risk stratification and inform extubation decisions, though external validation is necessary prior to its routine clinical application.

## Introduction

Neonatal respiratory distress syndrome (NRDS) is one of the leading causes of neonatal mortality, primarily attributed to pulmonary surfactant deficiency, which results in reduced lung compliance and alveolar collapse, particularly in preterm infants (1). Rapid identification of the underlying cause of respiratory distress and selection of an appropriate and effective treatment strategy are critical for patient prognosis (2). As one of the primary interventions for NRDS in neonates (3), mechanical ventilation can promptly improve oxygenation and ventilation. However, prolonged use may lead to complications such as ventilator-induced lung injury (VILI) and infections (4). Notably, VILI is a major risk factor for bronchopulmonary dysplasia (BPD) (5), the most prevalent chronic respiratory disease in neonates, which may exert lifelong health consequences and impose significant socioeconomic burdens. Therefore, optimal timing for extubation is of profound importance in mitigating treatment risks and complications.

In recent years, multiple studies have demonstrated that lung ultrasound (LUS), owing to its noninvasive, real-time, and radiation-free advantages, holds substantial value in NRDS diagnosis and extubation outcome prediction (6, 7), serving as a pivotal tool for guiding clinical weaning. However, some studies (1) have highlighted limitations in LUS for predicting extubation outcomes in infants with lower gestational age (GA), particularly in assessing the need for reintubation. These divergent findings arise because existing research predominantly focuses on static LUS scores, with insufficient exploration of the predictive efficacy of dynamic LUS trajectory changes.

To address this gap, this study integrated multiple parameters, including pre-extubation dynamic LUS trajectories, GA, partial pressure of oxygen (PaO_2_), and oxygenation index (OI), to construct a combined predictive model for optimizing extubation timing in preterm neonates with NRDS receiving mechanical ventilation. By focusing on LUS trajectory rather than a single time-point score, we aim to provide a more robust and clinically intuitive framework for extubation risk stratification. We hypothesize that dynamic LUS trajectories provide superior predictive value for extubation outcomes compared to static scores, particularly in preterm neonates with NRDS.

## Materials and methods

### Study design and population

This retrospective study analyzed 266 neonates admitted to the Department of Neonatology at Banan Hospital Affiliated to Chongqing Medical University between January 2020 and December 2024. The study was approved by the Ethics Committee of Banan Hospital Affiliated to Chongqing Medical University (Ethics Approval No.: BNLL-KY-2024-015). Due to its retrospective nature, the requirement for informed consent was waived, and all data were anonymized. The dataset was randomly divided into a training cohort (70%) and a validation cohort (30%) at a 7:3 ratio. The training cohort was used for model training, while the validation cohort was employed for performance evaluation.

*Inclusion criteria*: (1) Gestational age between 28 and 38 weeks, with a birth weight ≥1,000 g; (2) Diagnosis of neonatal respiratory distress syndrome (NRDS); (3) Requirement for mechanical ventilation within 24 h after birth. The gestational age range of 28–38 weeks was selected to ensure a relatively homogeneous NRDS population and the feasibility of completing standardized serial LUS assessments before extubation. In our clinical setting, extremely preterm infants (<28 weeks) rarely undergo planned extubation with sufficient stability to complete four pre-extubation LUS examinations, whereas infants >38 weeks seldom present with classical surfactant-deficient NRDS. Therefore, this range represents the main ventilated NRDS population in whom planned extubation and serial LUS assessment are routinely considered. *Exclusion criteria*: (1) Congenital heart disease, hypoxic–ischemic encephalopathy, other severe complications (e.g., intracranial hemorrhage, pulmonary hemorrhage, pulmonary infection, pleural effusion, pneumothorax), or missing critical clinical data; (2) Infants without complete four time-point pre-extubation LUS data were excluded to ensure integrity of trajectory classification.

Based on whether reintubation was required within 48 h after extubation, the neonates were classified into the extubation success group (*n* = 162) and the extubation failure group (*n* = 104). The timing of extubation and duration of mechanical ventilation were determined by attending physicians according to clinical manifestations and the 2022 European Consensus Guidelines on the Management of Respiratory Distress Syndrome (8). Data collection and analysis were conducted independently of the attending physicians to avoid potential bias.

### Sample size calculation

The sample size was calculated using the events per variable (EPV) method, a widely accepted statistical approach by Riley et al. (9). In our cohort, the estimated incidence of extubation failure was 0.3909, with an expected total of 4 independent variables and a training cohort proportion of 0.7. The required sample size was calculated as follows:


$$\text{Sample Size}=\frac{\text{Number of Variables}\times \mathrm{EPV}}{\text{IncidenceRate}}=\frac{4\times 10}{0.3909}=102.3$$

Training cohort (*n*) = 102.3 cases. Considering a 10% rate of invalid samples, the minimum total sample size required was 163 cases. Ultimately, 266 preterm neonates were included.

### Data collection

*General information*: gestational age, sex, birth weight, Apgar score, delivery mode, and duration of mechanical ventilation. Blood Gas Analysis: PaO_2_, PaCO_2_, pH, and oxygenation index $\left(\mathrm{OI}=\frac{\mathrm{MAP}\times \mathrm{Fi}O_{2}}{\mathrm{Pa}O_{2}}\times 100\right)$ . Echocardiography: Left ventricular ejection fraction (LVEF).

### Lung ultrasound score (LUS)

All enrolled neonates underwent LUS examinations at 48, 24, 12, and 2 h before extubation using a GE Logiq e portable Doppler ultrasound system with an 8–12 MHz linear probe. These scans were scheduled as part of our standardized pre-extubation assessment pathway. Each lung was divided into six regions—anterior, lateral, and posterior, each further split into upper and lower regions—resulting in a 12-regions assessment with a total possible score of 0–36 (Supplementary Figure S1). Examinations were performed in the supine and lateral positions while the neonates were calm. Scanned systematically from right to left, anterior to posterior, and superior to inferior, posterior regions imaging is also performed in the lateral decubitus position. Scans were performed at four time points (48, 24, 12, and 2 h before extubation) following a standardized sequence to minimize positional variability. Each zone received a score from 0 to 3 based on its worst observed finding, and these zone scores were summed to produce the total lung ultrasound score.

Scoring was performed according to Brat et al. (10), with each region assigned a score (0–36 points) based on the most severe ultrasound findings:

0 points: Normal aeration (A-lines only, with occasional B-lines).

1 point: Moderate reduction in aeration, interstitial syndrome, localized pulmonary edema (B-lines occupying <50% of the intercostal space), or subpleural consolidation.

2 points: Severe reduction in aeration, [lveolar edema (confluent B-lines occupying the entire intercostal space)].

3 points: Pulmonary consolidation.

The total examination time did not exceed 4 min. All LUS were performed by two ultrasound specialists with over 5 years of experience. Interobserver agreement was excellent (*κ* = 0.89) based on evaluation of 30 randomly selected scans. Assessors remained blinded to extubation outcomes throughout the scoring process.

### Definition of extubation failure

After extubation, the patient developed respiratory failure. Despite clinical interventions such as oxygen therapy, hypoxemia or respiratory distress symptoms were not effectively relieved, and mechanical ventilation had to be restarted within 48 h.

### Statistical analysis and model development

*Statistical methods*: data were analyzed using *R* (version 4.4.1). Continuous variables were expressed as mean ± standard deviation (normally distributed) or median (non-normally distributed), with group comparisons performed using one-way ANOVA or the Wilcoxon rank-sum test, respectively. Categorical variables were presented as frequencies (%), with group comparisons conducted using the chi-square test or Fisher’s exact test, as appropriate.

LUS trajectory analysis was performed using the lcmm package to identify heterogeneous LUS dynamic patterns. The optimal number of classes was selected based on BIC. Trajectory classes were derived in the training cohort and applied to the validation cohort, with infants assigned to the most likely class according to posterior probabilities.

Model development followed a standard pipeline: candidate variables were screened by univariate logistic regression (*p* < 0.10), selected by LASSO with 10-fold cross-validation (1-SE rule), and entered into a bidirectional stepwise multivariate logistic regression. A nomogram was constructed based on the final coefficients and internally validated using 1,000 bootstrap resamples. Discrimination, calibration, and clinical utility were evaluated using ROC-AUC, calibration curves with the Hosmer–Lemeshow test, and decision curve analysis, respectively. Model performance was assessed in the internal validation cohort. Analyses were conducted in R using glmnet, rms, pROC, ggplot2, and ggscidca. A two-sided *p* < 0.05 was considered statistically significant.

## Results

### LUS trajectory patterns

**Figure 1:**
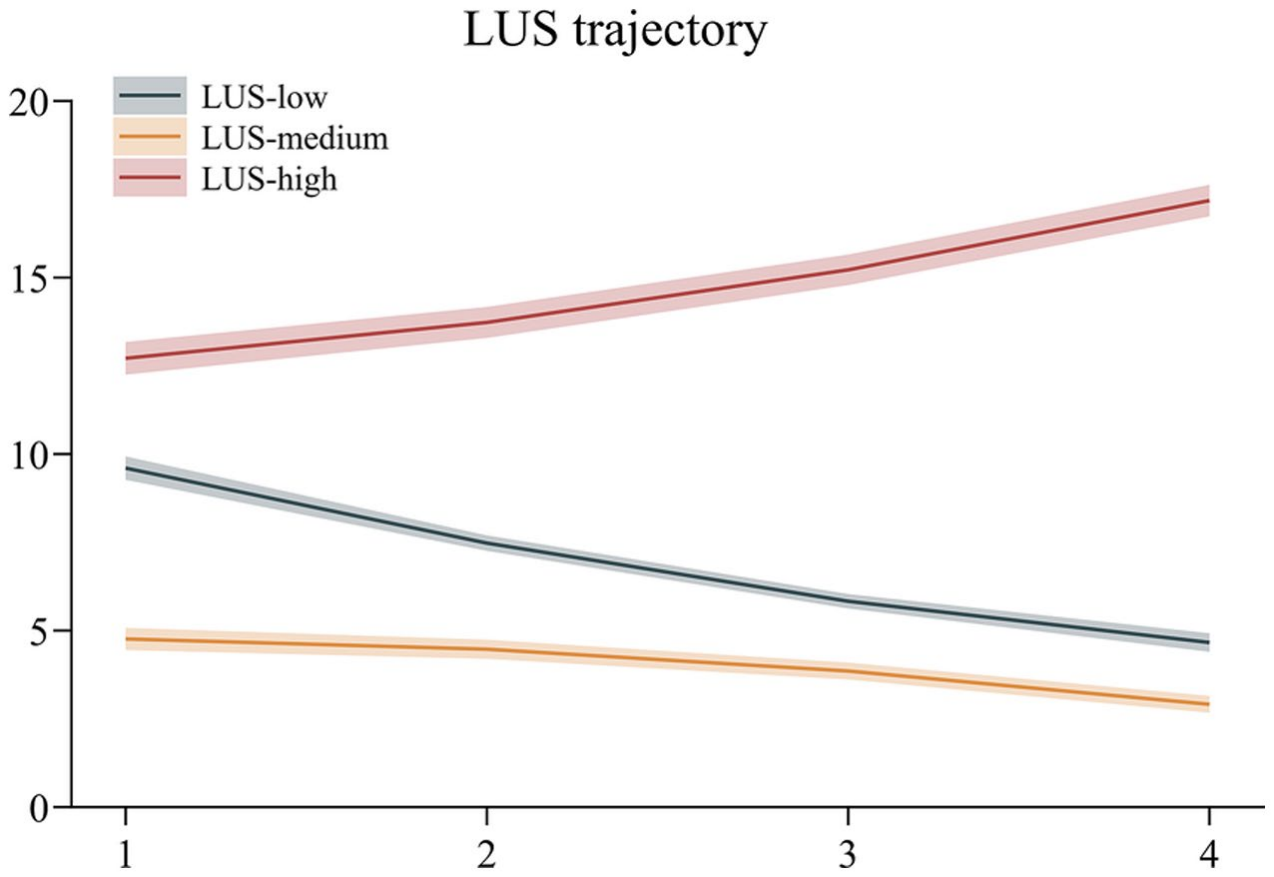
Trajectories of lung ultrasound score (LUS) over time. Lines indicate mean LUS trajectories and shaded bands represent 95% confidence intervals. Three trajectory groups were identified: LUS-high (high-growth), LUS-medium (moderate decline), and LUS-low (marked decline).

Figure 1 illustrates three distinct LUS evolution trajectories, with each trajectory line representing the mean trend of a potential class, and the shaded area indicating its 95% confidence interval. Based on the graphical patterns and LUS levels, the trajectories were designated as follows:

*High-increasing trajectory (LUS-high, red)*: This group exhibited the highest initial LUS score (approximately 12.5 points), with a persistently upward trend, suggesting potential deterioration in lung ultrasound scores over time.

*Low-decreasing trajectory (LUS-low, gray)*: Characterized by a moderately high initial score (approximately 9.5 points) and a steadily declining trend, indicating significant pulmonary improvement.

*Moderate-stable decreasing trajectory (LUS-medium, orange)*: Displayed the lowest initial score (approximately 4.8 points) with a gradual decline, reflecting an initially favorable condition and sustained improvement.

### Baseline characteristics

**Figure 2:**
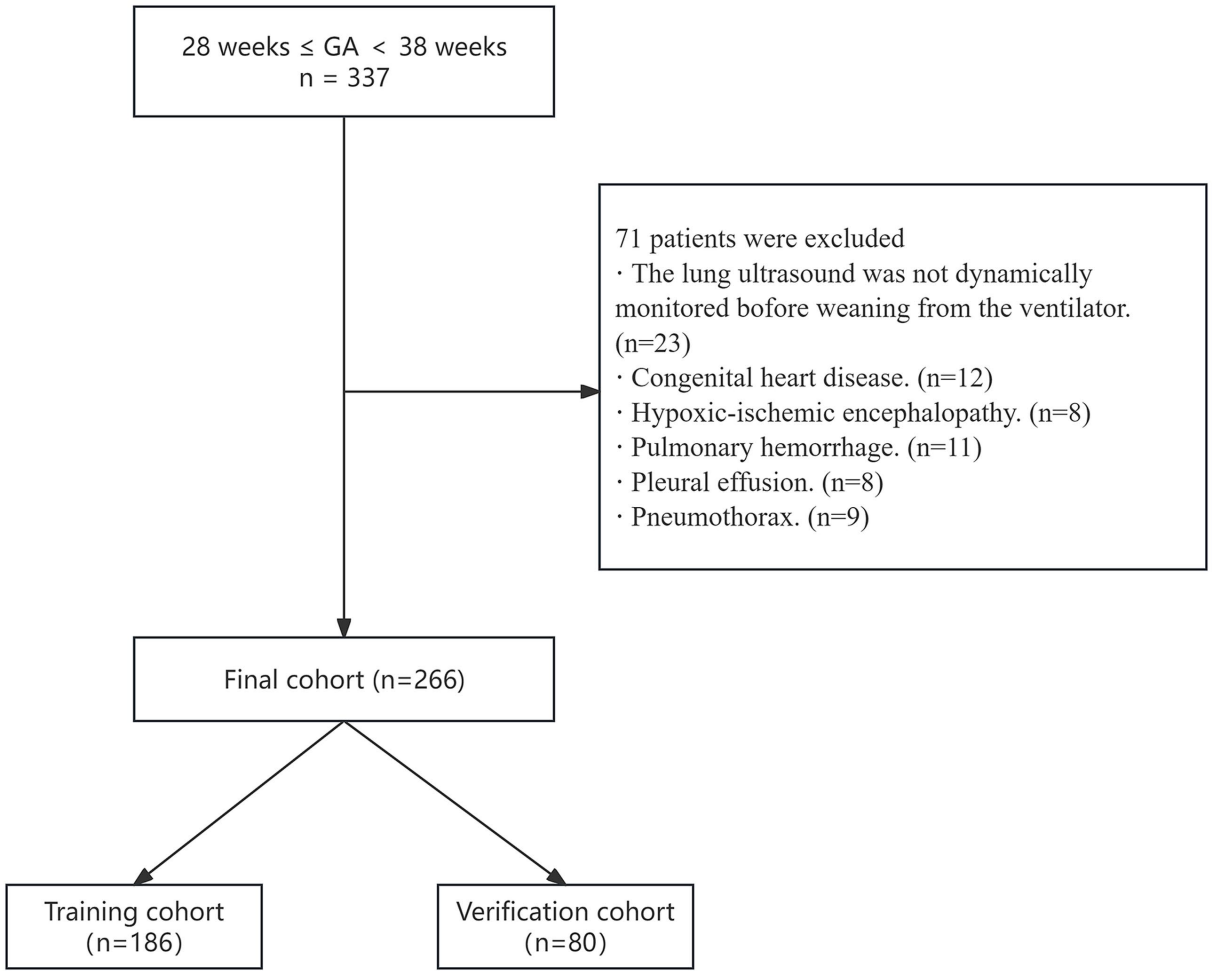
Flow chart of patient selection. Exclusions: lack of serial pre-extubation LUS assessments (*n* = 23), congenital heart disease (*n* = 12), hypoxic–ischemic encephalopathy (*n* = 8), pulmonary hemorrhage (*n* = 11), pleural effusion (*n* = 8), pneumothorax (*n* = 9).

A total of 337 infants were initially enrolled, with 71 excluded due to unmet inclusion criteria, leaving 266 infants for final analysis (Figure 2). Based on the need for reintubation within 48 h, the cohort was stratified into the successful extubation group (*n* = 162) and the failed extubation group (*n* = 104). Comparative analysis revealed significant differences in LUS trajectory distribution between the two groups (*χ*^2^ = 97.655, *p* < 0.001). The failed extubation group had a significantly higher proportion of LUS-high infants (61.5% vs. 6.2%), whereas the successful extubation group was predominantly composed of LUS-low (42.6% vs. 13.5%) and LUS-medium (51.2% vs. 25.0%) infants. The failed extubation group also exhibited lower gestational age (GA), birth weight, PaO_2_, and left ventricular ejection fraction (LVEF), along with longer mechanical ventilation duration and higher oxygenation index (OI) (Table 1).

**Table 1:** Baseline characteristics.

Characteristics	Success (*n* = 162)	Failure (*n* = 104)	Statistic	*p*
LUS trajectory, *n* (%)			97.655	<0.001
LUS-low	69 (42.6)	14 (13.5)		
LUS-medium	83 (51.2)	26 (25.0)		
LUS-high	10 (6.2)	64 (61.5)		
LUS_48 h, M (Q1, Q3)	7.00 (5.00, 9.00)	11.50 (10.00, 13.00)	−7.398	<0.001
LUS_24 h, M (Q1, Q3)	6.00 (4.25, 7.00)	12.00 (5.75, 14.00)	−5.837	<0.001
LUS_12 h, M (Q1, Q3)	5.00 (4.00, 6.00)	13.00 (5.00, 16.00)	−6.975	<0.001
LUS_2 h, M (Q1, Q3)	4.00 (3.00, 4.00)	15.00 (5.50, 18.00)	−8.652	<0.001
Sex, *n* (%)			0.391	0.532
Male	92 (56.8)	55 (52.9)		
Female	70 (43.2)	49 (47.1)		
Gestational age, M (Q1, Q3)	35.00 (33.14, 36.82)	30.43 (29.57, 34.14)	8.114	<0.001
Birth weight, M (Q1, Q3)	2.43 (1.94, 2.76)	2.03 (1.50, 2.51)	3.637	<0.001
Apgar score 1 min, M (Q1, Q3)	9.00 (8.00, 10.00)	9.00 (8.00, 10.00)	0.552	0.581
Apgar score 5 min, M (Q1, Q3)	9.00 (9.00, 10.00)	10.00 (9.00, 10.00)	−0.868	0.385
Mode of delivery, *n* (%)			0.160	0.690
Caesarean delivery	99 (61.1)	61 (58.7)		
Vaginal delivery	63 (38.9)	43 (41.3)		
Time of mechanical ventilation, M (Q1, Q3)	69.00 (39.00, 97.00)	72.50 (44.75, 117.25)	−1.531	0.126
PaO_2_, Mean ± SD	78.00 (71.00, 89.00)	60.50 (52.75, 67.00)	8.699	<0.001
PaCO_2_, M (Q1, Q3)	39.00 (34.00, 45.75)	41.00 (34.75, 46.00)	−1.075	0.282
pH, Mean ± SD	7.34 (7.29, 7.39)	7.35 (7.31, 7.38)	−0.982	0.326
OI, M (Q1, Q3)	7.10 (5.40, 8.00)	7.35 (6.40, 8.50)	−4.373	<0.001
Left ventricular ejection fraction, M (Q1, Q3)	69.00 (64.00, 73.00)	67.00 (57.00, 73.00)	2.604	0.009

SD, standard deviation, M, Median, Q1, 1st Quartile, Q3, 3rd Quartile, h, hours.

Continuous data presented as Mean ± SD (normally distributed) or M (Q1–Q3) (non-normally distributed).

Categorical data presented as *n* (%).

To validate consistency across datasets, patients were randomly divided into a training cohort (*n* = 186, 70%) and a validation cohort (*n* = 80, 30%). The baseline characteristics of all patients between the training and validation cohorts are presented in Table 2. In the training cohort, the failed extubation group again showed a significantly higher proportion of LUS-high infants (64.9% vs. 6.2%), while LUS-low and LUS-medium predominated in the successful extubation group (Supplementary Table S1). A similar trend was observed in the validation cohort, with the failed extubation group demonstrating a higher LUS-high proportion (53.3% vs. 6.0%) and the successful extubation group primarily comprising LUS-low and LUS-medium infants (Supplementary Table S2).

**Table 2:** Baseline characteristics of all patients between the training and validation cohorts.

Characteristics	Total (*n* = 266)	Training (*n* = 186)	Validation (*n* = 80)	Statistic	*p*
LUS trajectory, *n* (%)				1.510	0.470
LUS-low	83 (31.2)	59 (31.7)	24 (30.0)		
LUS-medium	109 (41.0)	72 (38.7)	37 (46.2)		
LUS-high	74 (27.8)	55 (29.6)	19 (23.8)		
LUS_48 h, M (Q1, Q3)	8.00 (5.00, 11.00)	9.00 (5.00, 11.00)	8.00 (5.00, 11.00)	0.701	0.483
LUS_24 h, M (Q1, Q3)	7.00 (5.00, 12.00)	7.00 (5.00, 12.00)	6.00 (5.00, 8.25)	1.266	0.206
LUS_12 h, M (Q1, Q3)	6.00 (4.00, 13.00)	6.00 (4.00, 13.00)	6.00 (4.00, 7.00)	1.120	0.263
LUS_2 h, M (Q1, Q3)	4.00 (3.00, 15.00)	4.00 (3.00, 15.00)	4.00 (3.00, 6.00)	1.047	0.295
Sex, *n* (%)				1.282	0.258
Male	147 (55.3)	107 (57.5)	40 (50.0)		
Female	119 (44.7)	79 (42.5)	40 (50.0)		
Gestational age, M (Q1, Q3)	34.00 (30.46, 36.14)	34.00 (30.43, 36.11)	34.07 (31.00, 36.46)	−0.845	0.398
Birth weight, M (Q1, Q3)	2.27 (1.83, 2.70)	2.22 (1.77, 2.68)	2.42 (2.01, 2.78)	−2.146	0.032
Apgar score 1 min, M (Q1, Q3)	9.00 (8.00, 10.00)	9.00 (8.00, 10.00)	9.00 (8.00, 10.00)	−0.201	0.841
Apgar score 5 min, M (Q1, Q3)	9.00 (9.00, 10.00)	9.00 (9.00, 10.00)	9.50 (9.00, 10.00)	−0.443	0.658
Mode of delivery, *n* (%)				0.058	0.810
Caesarean delivery	160 (60.2)	111 (59.7)	49 (61.3)		
Vaginal delivery	106 (39.8)	75 (40.3)	31 (38.8)		
Time of mechanical ventilation, M (Q1, Q3)	70.00 (42.25, 101.00)	69.00 (38.25, 102.00)	71.00 (44.00, 98.25)	−0.309	0.758
PaO_2_, Mean ± SD	72.00 (61.00, 87.00)	72.00 (61.00, 83.75)	73.50 (61.00, 88.00)	−0.406	0.685
PaCO_2_, M (Q1, Q3)	40.00 (34.00, 46.00)	40.00 (34.00, 46.00)	38.50 (32.75, 45.00)	0.895	0.371
pH, Mean ± SD	7.34 (7.29, 7.39)	7.34 (7.29, 7.38)	7.35 (7.31, 7.39)	−1.221	0.222
OI, M (Q1, Q3)	7.20 (5.50, 8.00)	7.20 (5.43, 8.20)	7.20 (6.07, 8.00)	0.189	0.850
Left ventricular ejection fraction, M (Q1, Q3)	69.00 (63.00, 73.00)	69.00 (63.00, 74.00)	67.00 (60.75, 72.00)	1.952	0.051

SD, standard deviation, M, Median, Q1, 1st Quartile, Q3, 3rd Quartile, h, hours.

Continuous data presented as Mean ± SD (normally distributed) or M (Q1–Q3) (non-normally distributed).

Categorical data presented as *n* (%).

### Predictor screening and logistic regression model construction

In the training cohort, univariate logistic regression identified LUS trajectory, GA, birth weight, PaO_2_, OI, and LVEF as potential outcome-associated factors (Supplementary Table S3). To mitigate multicollinearity, LASSO regression was applied using variables with *p* < 0.1. The optimal lambda value (0.07, determined by the one-standard-error rule) selected LUS trajectory, GA, PaO_2_, and OI as non-zero coefficient variables (Supplementary Figure S2). Subsequent multivariate logistic regression confirmed these as independent predictors, with GA and PaO_2_ as protective factors (OR < 1) and LUSclass and OI as risk factors (OR > 1) (Table 3).

**Table 3:** Multivariate logistic analysis.

Characteristics	OR (95%CI)	*p*
LUS trajectory		
LUS-low	Reference	
LUS-medium	6.676 (2.182, 23.826)	0.002
LUS-high	24.099 (7.520, 88.905)	<0.001
Gestational age	0.759 (0.627, 0.909)	0.003
PaO_2_	0.964 (0.931, 0.997)	0.033
OI	1.409 (1.120, 1.804)	0.005

OR, odds ratio; CI, confidence interval.

### Correlation heatmap of predictors

A heatmap of Pearson correlation coefficients among predictors LUS trajectory, GA, PaO_2_, and OI is presented in Supplementary Figure S3. Color intensity reflects correlation strength, with red indicating positive and blue indicating negative correlations. Results demonstrated low inter-predictor correlations, with no evidence of significant multicollinearity.

### Nomogram construction, predictive accuracy, and net benefit

**Figure 3:**
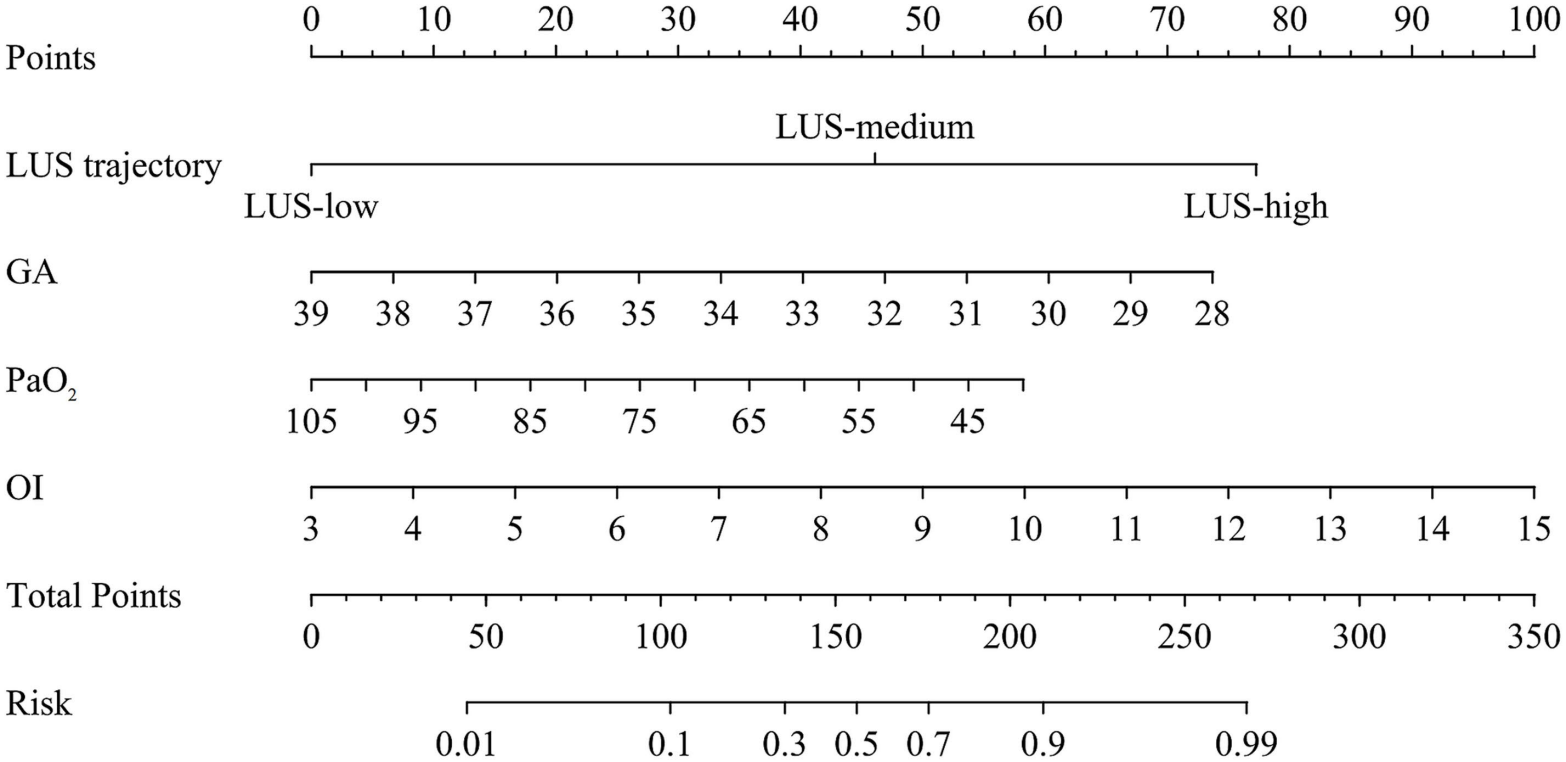
Nomogram for predicting extubation outcome in neonates with NRDS. Predictors include LUS trajectory group, gestational age, PaO_2_, and oxygenation index. Points are assigned for each predictor and summed to obtain a total score, which corresponds to the predicted probability of extubation outcome.

**Figure 4:**
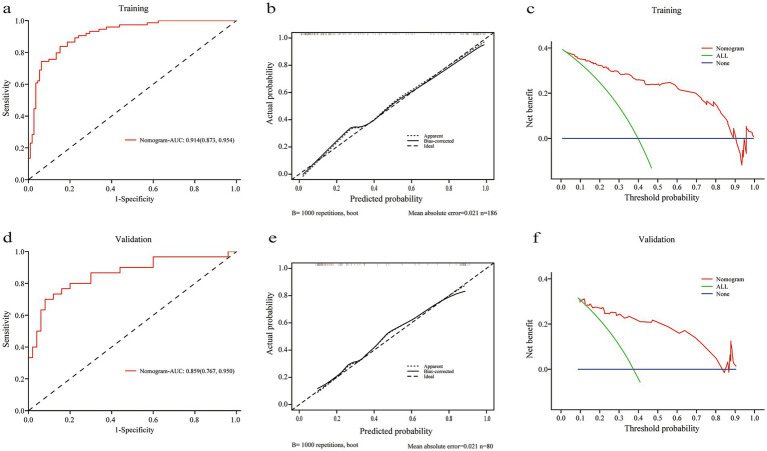
Model development and performance. **(a)** ROC curve in the development cohort (AUC 0.914, 95% CI: 0.873–0.954). **(b,c)** Calibration plots in the development and internal validation cohorts. **(d)** ROC curve in the internal validation cohort (AUC 0.859, 95% CI: 0.767–0.950). **(e,f)** Decision curve analysis in the development and internal validation cohorts.

A nomogram integrating these significant variables was developed (Figure 3), assigning scores to each predictor. The total score, derived from summing individual variable contributions, estimated extubation failure risk. The model demonstrated excellent predictive accuracy in the training cohort (AUC = 0.914, 95% CI: 0.873–0.954) (Figure 4a), with a calibration curve closely aligned with the ideal diagonal (Figure 4b). Decision curve analysis (DCA) revealed substantial net benefit, supporting its clinical utility (Figure 4c). Validation cohort performance remained robust (AUC = 0.859, 95% CI: 0.767–0.950) (Figure 4d), with similarly favorable calibration (Figure 4e) and DCA results (Figure 4f), further affirming its clinical applicability.

## Discussion

This study developed and validated a combined prediction model based on lung ultrasound score (LUS) trajectory to predict the weaning outcome of mechanical ventilation in neonates with respiratory distress syndrome (NRDS). The results demonstrated that LUS trajectory, gestational age, partial pressure of oxygen (PaO_2_), and oxygenation index (OI) were key predictors of successful weaning in neonates with NRDS. This study does not aim to replace established extubation criteria. Instead, we propose LUS trajectory assessment as an adjunctive, objective tool that may be particularly useful when clinical signs are borderline or when clinicians face uncertainty regarding extubation readiness. Most previous studies focused on static pre-extubation LUS measurements. Our findings suggest that trajectory-based assessment captures the evolution of lung aeration over time and may identify infants who show early improvement but subsequently plateau or deteriorate near planned extubation. This dynamic information provides a clinically intuitive risk stratification framework, which may explain the strong association between the high-increasing trajectory and extubation failure.

Neonatal respiratory distress syndrome (NRDS) is one of the most common conditions in neonatal intensive care (3). Mechanical ventilation is a critical intervention for NRDS, as it rapidly improves pulmonary ventilation and alleviates respiratory distress (11). However, prolonged mechanical ventilation may lead to complications such as airway injury, ventilator-induced lung injury, and infection (25). Therefore, the timing of weaning is crucial. The fundamental cause of weaning failure lies in the inability of neonates to maintain adequate spontaneous ventilation after discontinuation of mechanical support. Some scholars suggest that NRDS is associated with a deficiency or insufficient synthesis of pulmonary surfactant, which reduces lung compliance and leads to alveolar collapse (12).

In recent years, LUS has been increasingly applied in the diagnosis of NRDS and the prediction of optimal weaning timing (13). In the study by Li et al. (7), LUS prior to weaning was significantly correlated with OI and arterial blood gas parameters. The LUS in the successful weaning group was significantly lower than that in the failure group (5 vs. 12.5, *p* < 0.001), indicating that LUS could serve as a sensitive and accurate predictor of successful weaning. However, Sett et al. (1) noted certain limitations of LUS in predicting outcomes for infants with a gestational age <32 weeks, particularly in determining the need for reintubation. We hypothesize that this may be related to the lack of dynamic monitoring of pre-weaning pulmonary morphological changes. Currently, studies investigating the predictive value of pre-weaning LUS trajectory changes for weaning outcomes remain limited. Our study identified the pre-weaning LUS trajectory as a predictor of weaning outcomes in neonates with NRDS undergoing mechanical ventilation. Using the LUS trajectory model, neonates were stratified into high-risk (LUS-high), intermediate-risk (LUS-medium), and low-risk (LUS-low) groups. The high-risk group exhibited a 24-fold increased risk of weaning failure compared to the low-risk group (OR = 24.099, 95% CI 7.520–88.905, *p* < 0.001), while the intermediate-risk group had a 6.7-fold higher risk (OR = 6.676, 95% CI 2.182–23.826, *p* = 0.002). These findings underscore the profound value of LUS trajectory in assessing disease severity and predicting adverse outcomes, significantly enhancing the accuracy of weaning decisions. The high-increasing LUS trajectory likely reflects progressive alveolar collapse and interstitial edema, indicative of persistent surfactant dysfunction and impaired lung compliance. Therefore, we recommend incorporating LUS trajectory changes into weaning assessments. In patients with sustained LUS improvement, confidence in weaning may be higher, whereas those with unfavorable LUS trajectories should be closely monitored, with proactive management of potential complications (e.g., ventilator-induced lung injury, infection) to avoid premature or inappropriate weaning.

Gestational age is a critical marker of neonatal lung maturity. Immature lung development predisposes neonates to pulmonary surfactant deficiency, reduced lung compliance, and abnormal pulmonary vascular development, thereby increasing the risk of NRDS (14, 15). Bronchopulmonary dysplasia (BPD) is one of the most common adverse outcomes (16). Zhong et al. (17) reported that lower gestational age was associated with a higher incidence of BPD (51.7% in neonates <28 weeks). In the study by Tao et al. (18), 102 of 625 neonates with NRDS (16.3%) developed BPD, confirming BPD as a risk factor for NRDS. Zhao et al. (19) observed that the incidence of NRDS decreased with increasing gestational age. These studies collectively highlight gestational age as a significant determinant of BPD and NRDS. Additionally, Yue et al. (20) demonstrated that gestational age independently correlated with the likelihood of mechanical ventilation. In our study, gestational age also predicted weaning outcomes, with each additional week of gestation reducing the risk of weaning failure by approximately 24.1% (OR = 0.759, 95% CI 0.627–0.909, *p* = 0.003). This finding emphasizes the importance of gestational age as a simple and readily available predictive marker.

Partial pressure of oxygen (PaO_2_) is a core parameter for evaluating pulmonary gas exchange (21). A lower PaO_2_ reflects potential oxygenation impairment under current ventilatory support (22), suggesting possible post-weaning oxygenation deterioration. Our study confirmed that pre-weaning PaO_2_ was a key predictor, with each 1 mmHg increase in PaO_2_ reducing the risk of weaning failure by approximately 3.6% (OR = 0.964, 95% CI 0.931–0.997, *p* = 0.033). Although PaO_2_ is routinely assessed before weaning, its predictive performance was inferior to LUS trajectory changes. Thus, PaO_2_ should be integrated with LUS trajectory and other indicators rather than relied upon in isolation.

The oxygenation index (OI), which combines fractional inspired oxygen (FiO_2_), mean airway pressure (MAP), and PaO_2_, provides a comprehensive assessment of the severity of oxygenation dysfunction and indicates the need for mechanical ventilatory support (23). An elevated OI suggests higher respiratory support requirements and potential inadequacy of spontaneous oxygenation maintenance (24). Our study demonstrated that each 1-unit increase in OI raised the risk of weaning failure by approximately 40.9% (OR = 1.409, 95% CI 1.120–1.804, *p* = 0.005). This result aligns with LUS trajectory findings, further supporting the role of OI in weaning risk assessment.

This study not only analyzed LUS from a dynamic trajectory perspective but also constructed a combined prediction model incorporating gestational age, PaO_2_, and OI. This model effectively overcomes the limitations of single parameters, significantly improving the accuracy of predicting weaning outcomes in neonates undergoing mechanical ventilation. The Hosmer–Lemeshow test indicated good model fit (*p* = 0.624), with the calibration curve closely approximating the ideal diagonal. Decision curve analysis further demonstrated the model’s superior net benefit. Validation cohort results confirmed the robustness and reliability of the predictive nomogram. Clinically, this model may facilitate early identification of high-risk neonates, optimizing weaning timing and strategies while reducing unnecessary weaning attempts and associated complications.

However, this study has several limitations. Because this was a single-center retrospective study with internal validation only, the model’s generalizability remains uncertain. This study lacks data on certain clinical confounding factors, including surfactant dosage and timing, ventilator settings prior to extubation, and the type of noninvasive support used afterward; these omissions may affect the model’s stability. We acknowledge concerns regarding the feasibility of four serial LUS examinations and the potential for introducing bias. In our unit, each examination required less than 4 min and was integrated into routine bedside evaluation, making serial assessment operationally feasible. Nevertheless, we do not suggest that four scans should be mandatory in all NICUs. Our trajectory-based framework provides a proof of concept, but future external validation in multicenter cohorts is required before routine clinical use.

## Conclusion

Integrating dynamic LUS trajectories with established clinical markers offers a non-invasive, real-time method for assessing extubation readiness in neonates with NRDS. Although internally validated, the model requires prospective external evaluation to confirm its generalizability and effect on clinical outcomes.

## Data Availability

The original contributions presented in the study are included in the article/Supplementary material, further inquiries can be directed to the corresponding authors.

## Supplementary material

SUPPLEMENTARY FIGURE S1 Schematic figure of lung regional segmentation.

SUPPLEMENTARY FIGURE S2 LASSO coefficient paths. Ten-fold cross-validation curve [binomial deviance vs log(*λ*)] used to select the optimal *λ*.

SUPPLEMENTARY FIGURE S3 Pearson correlation heatmap of predictors (LUS class, gestational age, PaO_2_, oxygenation index).
