# Supplementary figures and images for: Application of a combined predictive model based on lung ultrasound score trajectory changes in deciding mechanical ventilator weaning for neonatal respiratory distress syndrome: a retrospective study
Source: Front Med (Lausanne). 2026 Mar 11;13:1764757. doi: 10.3389/fmed.2026.1764757 (PMC13013512; doi:10.3389/fmed.2026.1764757)

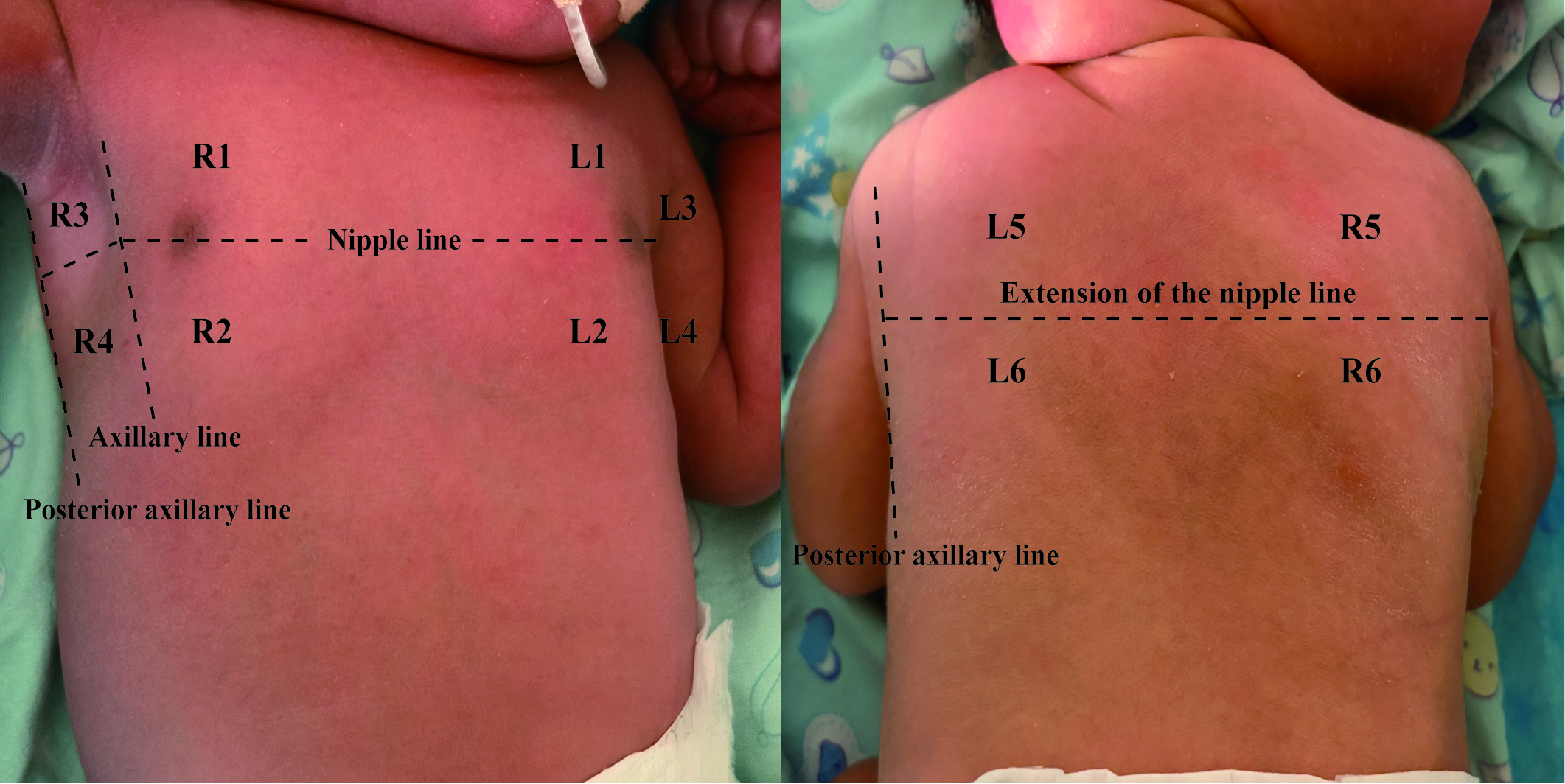

Supplement: SUPPLEMENTARY FIGURE S1 — Schematic figure of lung regional segmentation. [file Image_1.TIF]

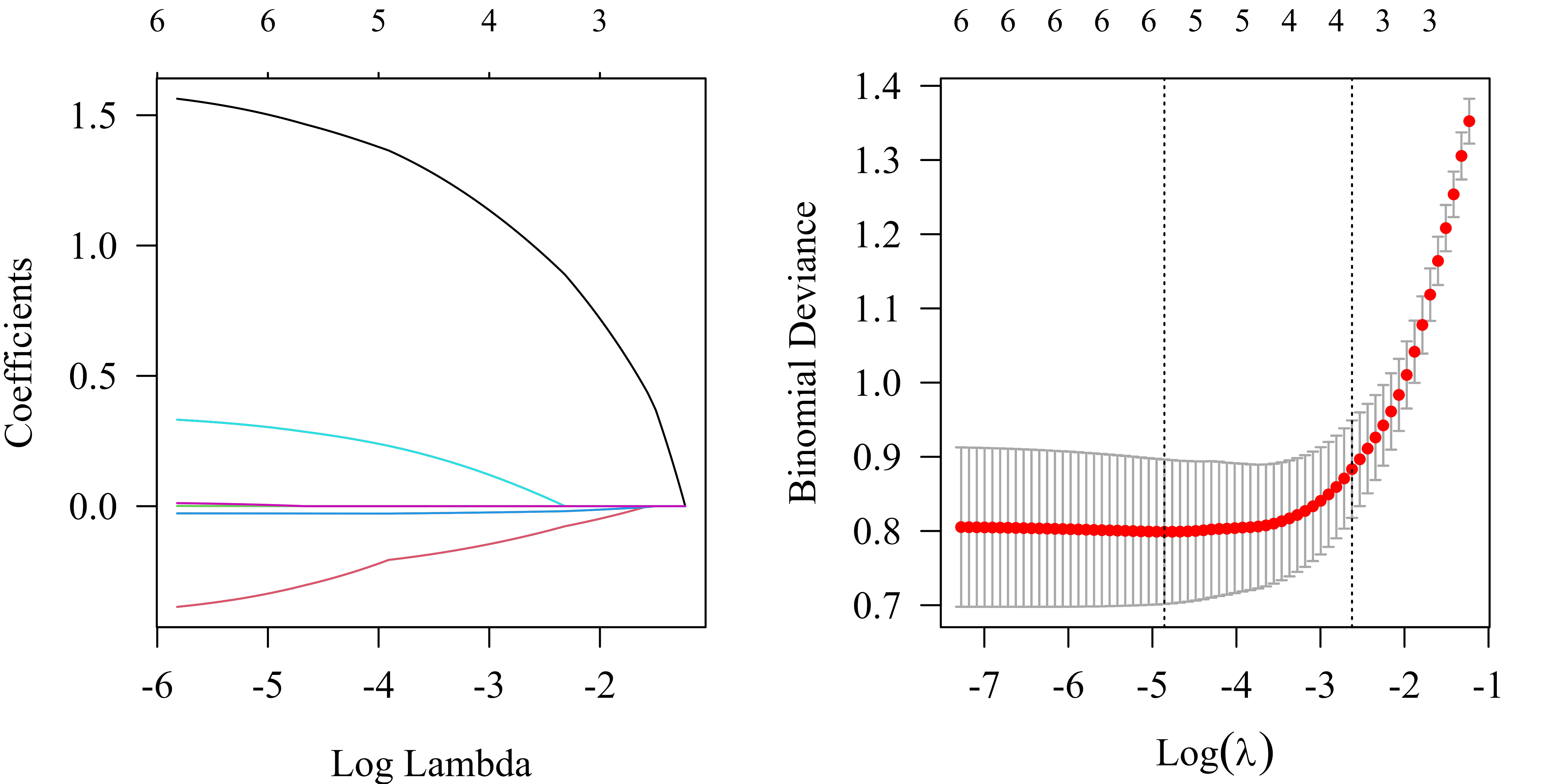

Supplement: SUPPLEMENTARY FIGURE S2 — LASSO coefficient paths. Ten-fold cross-validation curve [binomial deviance vs log(λ)] used to select the optimal λ. [file Image_2.TIF]

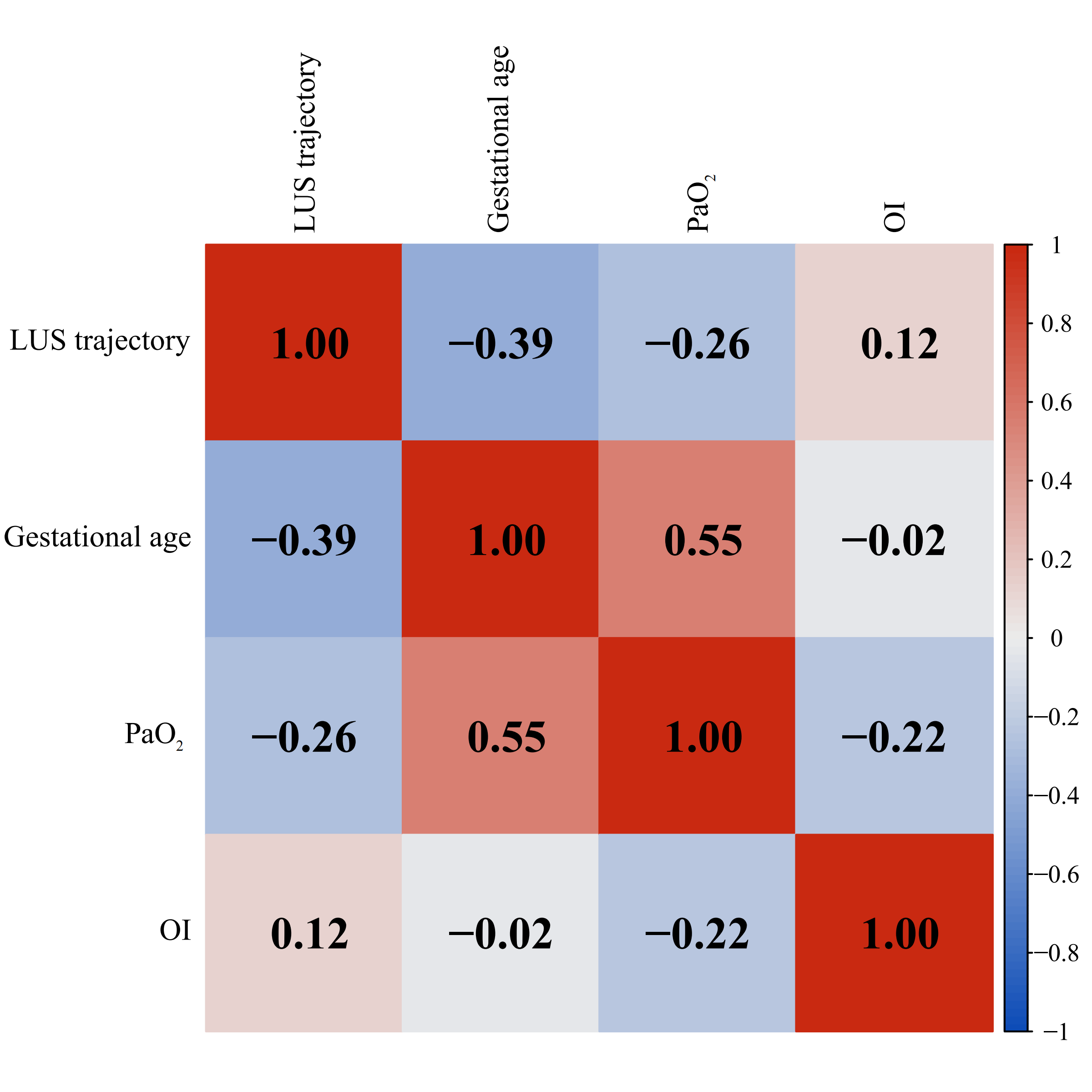

Supplement: SUPPLEMENTARY FIGURE S3 — Pearson correlation heatmap of predictors (LUS class, gestational age, PaO2, oxygenation index). [file Image_3.TIF]
