# Supplementary material for: Application of a combined predictive model based on lung ultrasound score trajectory changes in deciding mechanical ventilator weaning for neonatal respiratory distress syndrome: a retrospective study
Source: Front Med (Lausanne). 2026 Mar 11;13:1764757. doi: 10.3389/fmed.2026.1764757 (PMC13013512; doi:10.3389/fmed.2026.1764757)
Supplement: Supplementary file 4 [file Table_1.DOCX]

**Table S1. Baseline characteristics-Training**

| Characteristics | Success (n = 112) | Failure (n = 74) | statistic | P |
| --- | --- | --- | --- | --- |
| LUS trajectory, n (%) |  |  | 76.365 | < 0.001 |
| -LUS-low | 52 (46.4) | 7 (9.5) |  |  |
| -LUS-medium | 53 (47.3) | 19 (25.7) |  |  |
| -LUS-high | 7 (6.2) | 48 (64.9) |  |  |
| LUS_48h, M (Q1,Q3) | 8.00 (5.00, 9.00) | 12.00 (8.00, 13.00) | -6.022 | < 0.001 |
| LUS_24h, M (Q1,Q3) | 6.50 (4.00, 8.00) | 12.00 (5.25, 14.00) | -5.116 | < 0.001 |
| LUS_12h, M (Q1,Q3) | 6.00 (4.00, 6.00) | 13.00 (5.00, 16.00) | -5.965 | < 0.001 |
| LUS_2h M (Q1,Q3) | 4.00 (3.00, 4.00) | 15.00 (6.00, 18.00) | -7.297 | < 0.001 |
| Sex, n(%) |  |  | 0.030 | 0.863 |
| -Male | 65 (58.0) | 42 (56.8) |  |  |
| -Female | 47 (42.0) | 32 (43.2) |  |  |
| Gestational age, M (Q1,Q3) | 35.07 (32.86, 36.71) | 30.29 (29.57, 34.11) | 6.874 | < 0.001 |
| Birth weight, M (Q1,Q3) | 2.38 (1.85, 2.75) | 2.01 (1.47, 2.50) | 3.078 | 0.002 |
| Apgar score 1min, M (Q1,Q3) | 9.00 (8.00, 10.00) | 9.00 (8.00, 10.00) | 0.794 | 0.427 |
| Apgar score 5min, M (Q1,Q3) | 9.00 (9.00, 10.00) | 10.00 (9.00, 10.00) | -0.905 | 0.365 |
| Mode of delivery, n (%) |  |  | 0.066 | 0.798 |
| -Caesarean delivery | 66 (58.9) | 45 (60.8) |  |  |
| -Vaginal delivery | 46 (41.1) | 29 (39.2) |  |  |
| Time of mechanical ventilation, M (Q1,Q3) | 60.50 (37.00, 93.00) | 93.00 (45.25, 133.50) | -2.727 | 0.006 |
| PaO_2_, Mean ± SD | 78.00 (71.00, 89.00) | 60.50 (53.00, 66.75) | 7.356 | < 0.001 |
| PaCO_2_, M (Q1,Q3) | 39.00 (34.00, 46.00) | 42.00 (35.00, 46.00) | -1.264 | 0.206 |
| pH, Mean ± SD | 7.33 ± 0.08 | 7.35 ± 0.06 | -1.608 | 0.110 |
| OI, M (Q1,Q3) | 7.10 (5.40, 8.00) | 8.00 (6.65, 8.50) | -4.076 | < 0.001 |
| Left ventricular ejection fraction, M (Q1,Q3) | 70.00 (64.75, 74.00) | 69.00 (58.25, 73.75) | 1.490 | 0.136 |
| **Abbreviations:** SD: standard deviation, M: Median, Q1: 1st Quartile, Q3: 3rd Quartile, h: hours | | | | |
| **Notes:**  Continuous data presented as Mean ± SD (normally distributed) or M (Q1–Q3) (non-normally distributed).  Categorical data presented as n (%). | | | | |
