# Supplementary material for: Application of a combined predictive model based on lung ultrasound score trajectory changes in deciding mechanical ventilator weaning for neonatal respiratory distress syndrome: a retrospective study
Source: Front Med (Lausanne). 2026 Mar 11;13:1764757. doi: 10.3389/fmed.2026.1764757 (PMC13013512; doi:10.3389/fmed.2026.1764757)
Supplement: Supplementary file 5 [file Table_2.DOCX]

**Table S2. Baseline characteristics-Validation**

| Characteristics | Success (n = 50) | Failure (n = 30) | statistic | P |
| --- | --- | --- | --- | --- |
| LUS trajectory, n (%) |  |  | 23.849 | < 0.001 |
| -LUS-low | 17 (34.0) | 7 (23.3) |  |  |
| -LUS-medium | 30 (60.0) | 7 (23.3) |  |  |
| -LUS-high | 3 (6.0) | 16 (53.3) |  |  |
| LUS_48h, M (Q1,Q3) | 6.00 (5.00, 8.00) | 11.00 (11.00, 13.00) | -4.293 | < 0.001 |
| LUS_24h, M (Q1,Q3) | 6.00 (5.00, 7.00) | 11.00 (6.00, 13.00) | -2.934 | 0.003 |
| LUS_12h, M (Q1,Q3) | 5.00 (4.00, 6.00) | 10.00 (5.25, 13.00) | -3.664 | < 0.001 |
| LUS_2h, M (Q1,Q3) | 4.00 (3.00, 4.00) | 15.00 (4.50, 15.75) | -4.621 | < 0.001 |
| Sex, n(%) |  |  | 0.853 | 0.356 |
| -Male | 27 (54.0) | 13 (43.3) |  |  |
| -Female | 23 (46.0) | 17 (56.7) |  |  |
| Gestational age, M (Q1,Q3) | 34.71 (33.61, 37.00) | 30.43 (29.64, 34.61) | 4.320 | < 0.001 |
| Birth weight, M (Q1,Q3) | 2.46 ± 0.52 | 2.21 ± 0.60 | 1.914 | 0.061 |
| Apgar score 1min, M (Q1,Q3) | 9.00 (8.00, 10.00) | 9.00 (8.00, 9.75) | -0.258 | 0.797 |
| Apgar score 5min, M (Q1,Q3) | 9.50 (9.00, 10.00) | 9.50 (9.00, 10.00) | -0.162 | 0.871 |
| Mode of delivery, n (%) |  |  | 1.268 | 0.260 |
| -Caesarean delivery | 33 (66.0) | 16 (53.3) |  |  |
| -Vaginal delivery | 17 (34.0) | 14 (46.7) |  |  |
| Time of mechanical ventilation, M (Q1,Q3) | 75.50 (44.00, 107.50) | 61.50 (44.50, 84.25) | 1.536 | 0.125 |
| PaO_2_, Mean ± SD | 80.94 ± 15.25 | 62.23 ± 17.66 | 4.823 | < 0.001 |
| PaCO_2_, M (Q1,Q3) | 39.46 ± 8.07 | 39.60 ± 9.25 | -0.069 | 0.945 |
| pH, Mean ± SD | 7.35 ± 0.07 | 7.34 ± 0.08 | 0.200 | 0.843 |
| OI, M (Q1,Q3) | 7.10 (5.62, 8.00) | 7.20 (6.35, 8.15) | -1.663 | 0.096 |
| Left ventricular ejection fraction, M (Q1,Q3) | 67.96 ± 6.31 | 63.20 ± 8.26 | 2.715 | 0.009 |
| **Abbreviations:** SD: standard deviation, M: Median, Q1: 1st Quartile, Q3: 3rd Quartile, h: hours | | | | |
| **Notes:**  Continuous data presented as Mean ± SD (normally distributed) or M (Q1–Q3) (non-normally distributed).  Categorical data presented as n (%). | | | | |
