# Supplementary material for: Application of a combined predictive model based on lung ultrasound score trajectory changes in deciding mechanical ventilator weaning for neonatal respiratory distress syndrome: a retrospective study
Source: Front Med (Lausanne). 2026 Mar 11;13:1764757. doi: 10.3389/fmed.2026.1764757 (PMC13013512; doi:10.3389/fmed.2026.1764757)
Supplement: Supplementary file 6 [file Table_3.DOCX]

**Table S3. Univariate Logistic analysis**

| Characteristics | OR(95%CI) | P |
| --- | --- | --- |
| LUS trajectory |  |  |
| -LUS-low | reference |  |
| -LUS-medium | 2.663 (1.072, 7.306) | 0.043 |
| -LUS-high | 50.939 (17.859, 170.262) | <0.001 |
| Sex |  |  |
| -Male | reference |  |
| -Female | 1.054 (0.580, 1.908) | 0.863 |
| Gestational age | 0.660 (0.577, 0.747) | <0.001 |
| Birth weight | 0.999 (0.999, 1.000) | 0.003 |
| Apgar score 1min | 0.913 (0.768, 1.083) | 0.295 |
| Apgar score 5min | 1.120 (0.841, 1.517) | 0.448 |
| Mode of delivery |  |  |
| -Caesarean delivery | reference |  |
| -Vaginal delivery | 0.925 (0.505, 1.681) | 0.798 |
| Time of mechanical ventilation | 1.001 (0.999, 1.003) | 0.231 |
| PaO_2_ | 0.924 (0.898, 0.946) | <0.001 |
| PaCO_2_ | 1.009 (0.979, 1.039) | 0.569 |
| pH | 23.542 (0.427, 1524.415) | 0.128 |
| OI | 1.386 (1.177, 1.670) | <0.001 |
| Left ventricular ejection fraction | 0.955 (0.914, 0.995) | 0.031 |
| **Abbreviations:** OR = odds ratio; CI = confidence interval; | | |
